# A genome-wide expression profile analysis reveals active genes and pathways coping with phosphate starvation in soybean

**DOI:** 10.1186/s12864-016-2558-9

**Published:** 2016-03-05

**Authors:** Qing Wang, Jiao Wang, Yuming Yang, Wenkai Du, Dan Zhang, Deyue Yu, Hao Cheng

**Affiliations:** National Center for Soybean Improvement, National Key Laboratory of Crop Genetics and Germplasm Enhancement, Nanjing Agricultural University, Nanjing, China; Collaborative Innovation Center of Henan Grain Crops, College of Agronomy, Henan Agricultural University, Zhengzhou, 450002 China

**Keywords:** Soybean, Root system, Low-P stress, Phosphorus deficiency, Expression profile

## Abstract

**Background:**

Phosphorus is one of the most important macronutrients that is required for plant growth and development. However, stress under low-P conditions has become a limiting factor that affects crop yields and qualities. Plants have developed strategies to cope with this, while few genes associated with low-P tolerance have been identified in soybean.

**Results:**

Genome-wide analyses were performed on the roots and leaves of a low-P-tolerant accession and a low-P-sensitive accession which were identified by hydroponic experiments under different P treatments. Through comparative analyses on the differently expressed genes, we explored 42 common genes that were highly correlated to low-P stress. The functional classification of these genes revealed 24 Gene Ontology (GO) terms of biological process including response to oxidation reduction, hormone stimuli, and biotic and abiotic stimuli. Additionally, three common pathways were identified.

**Conclusions:**

These results could not only promote the work on the molecular regulation mechanism under low-P stress in soybean, but also facilitate the cultivation of high-phosphorus-acquisition and high-phosphorus-utilization soybean varieties.

**Electronic supplementary material:**

The online version of this article (doi:10.1186/s12864-016-2558-9) contains supplementary material, which is available to authorized users.

## Background

As an essential constituent of many important compounds that are required for the development and growth of plants, phosphorus (P) actively participates in photosynthesis, respiration, carbohydrate metabolism, energy transduction and other processes [1]. In many agricultural systems, however, the concentration of absorbable phosphorus in soil is insufficient to ensure plant productivity [2].

As one of the most important grain and industrial crops, soybean (*Glycine max* L. Merrill) is a vital source of protein for human and animal food. However, soybean growth and yield are limited by varieties of edaphic factors, especially low phosphorus availability in soils [3]. Under low-P stress, soybean plants become dwarfed, leaf area is reduced, and necrotic spots might appear on lower leaves [4]. Also, flowering time is increased and the number of pods could decrease during the fruiting period [5]. Due to these P-mediated phenotypic effects on soybeans, phosphate fertilizers are used to overcome undesirable potential yield outcomes. Large quantities of phosphate fertilizer may cause environmental pollution as it is easily immobilized in the soil to form chelates or precipitates [6]. In order to alleviate the effects of phosphorus deficiency on soybean growth, it is particularly important for us to search for P-efficient soybean materials and investigate high-phosphorus-efficient genes.

Recently, four major groups of genes induced by low-P stress were reported [7]. One group was involved in sensing low-P signals, including local phosphate sensing that was proposed to occur in the primary root in *Arabidopsis thaliana* [8], and long-distance phosphate signaling which depends on the activity of transcription factors, such as *PHR1*, *PHL1* [9]. Members of another group mediated phosphate distribution by involving an internal regulation of phosphorus homeostasis, including SPX family and PHO1 that contains SPX domain in *Arabidopsis* [10]. The remaining two groups of genes mainly focus on the acquisition and transportation of phosphate. For example, *GmACP1* encodes an acid phosphatase, the overexpression of which in roots could increase the absorption of phosphate in the nutrient solution [11]. The PHT1 family consisting of 15 *PHT1* genes in soybean encoded phosphate transporters was demonstrated to be involved in not only directing phosphate uptake in roots, but also transporting phosphorus as phosphate [12, 13]. Generally, the acquisition and transportation of phosphate greatly depends on the root system as root morphology changes under P-deficient conditions to sustain plant growth. This includes an increase in number and length of lateral roots in maize (*Zea mays* L.) [14], longer and greater number of root hairs in low-P tolerant accessions of barley (*Hordeum vulgare* L*.*) and *Arabidopsis* [15, 16], and relatively longer primary roots in rice (*Oryza sativa* L.) when a low-phosphorus-related transcription factor *OsMYB2P-1* is over expressed [17]. Meanwhile, QTL (quantitative trait loci) mapping associated with soybean phosphorus efficiency have made processes. QTLs related to seed phosphorus content [18], phosphorus efficiency relevant root traits [19], and soybean tolerance to low phosphorus stress based on flower and pod abscission rate [20] have been identified. Furthermore, screening of soybean P-efficient materials identified P-efficient genotypes, such as HP119, HP134, Huaxai 1, Huaxia 2 etc. [21, 22].

As an abiotic stress, low-P conditions may induce a series of complex responses. It was demonstrated that phytohormone signaling pathways responded strongly to phosphate deprivation [23, 24]. The expression of a large set of genes associated with plant hormone signaling either induced or repressed root growth in *Arabidopsis* and *Zea mays* when suffering low-P conditions [23, 24]. In addition, changes of the secondary metabolites to low-P stress were shown with an accumulation of anthocyanin under long-term phosphorus deficiency [23]. Similar results were observed in *Zea mays* with genes related to the phenylpropanoid pathway identified as up-regulated or down-regulated under P starvation [24].

A large set of genes and signaling pathways that were associated with low-P stress had been uncovered using the technique of gene expression profiles and transcriptome analyses in *Arabidopsis*, rice, and *Zea mays* [23–27]. Recently, Zeng et al. identified phosphate-deficiency-responsive genes in soybean (*Glycine max* var. Williams 82) roots by high-throughput sequencing [28]. Selection of representative materials and consideration of different tissues will further reveal genes and pathways associated with low-P stress. Here, we selected a low-P-tolerant accession Chundou (CD) and a low-P-sensitive accession Yunhefengwodou (YH) from 219 soybean accessions through hydroponic experiments. Then microarray chips were performed on the roots and leaves of these two accessions. By analyzing the gene expression data, we identified 42 candidate genes and three common pathways that were induced by low-P stress. Our study provides candidate loci for functional identification of high-phosphorus-efficient genes which may be of great significance for cultivating P-efficient soybean accessions.

## Results

### Identification of low-P-tolerant accession CD and low-P-sensitive accession YH

With hydroponic experiments lasting for 10 days under different P treated conditions of 219 soybean accessions [29], we found that the soybean accession CD and YH showed similar root morphology under + P treated condition (Hoagland with 1.0 mmol/L P), while showing obvious morphology differences under -P treated condition (Hoagland with 0.01 mmol/L P). We then identified accession CD as a low-P-tolerant accession, and accession YH as a low-P-sensitive accession.

Then, we carried out a gradient experiment using full, half and quarter Hoagland’s solution and found that the + P and -P treated soybean plants showed most obvious morphology difference when grown in half Hoagland. Hence half Hoagland with 0.5 mmol/L P and 0.005 mmol/L P were considered as normal and low P treated conditions, respectively. The phenotypic traits of CD and YH are shown in Additional file 1: Figure S1. Primary root length was longer in CD than that in YH. The number of root hairs and lateral roots of CD were much more than those in YH. Microarray experiments were performed on the roots and leaves of CD and YH.

### Significantly differentially expressed genes in different P treated conditions

Under low P condition, 257 and 11 up-regulated differently expressed genes (DEGs) were found in the roots and leaves of low-P-tolerant accession CD respectively; while no up-regulated DEGs were found in the roots and leaves of low-P-sensitive accession YH compared with low-P treatment. For down-regulated DEGs, only 41 DEGs could be found in the roots of CD, 3 DEGs in the roots, and 7 DEGs in the leaves of YH (Table 1).

**Table 1:** The number of DEGs in different P treated conditions

Materials	Tissues	Number of up-regulated genes	Number of down-regulated genes	Up-regulated fold change	Down-regulated fold change
CD	Roots	257	41	2.00–33.78	0.11–0.49
	Leaves	11	0	2.32–5.29	--
YH	Roots	0	3	--	0.30–0.44
	Leaves	0	7	--	0.038–0.34

Generally, the number of DEGs in CD was more than that in YH, not only in the roots but also in the leaves (Table 1), which suggested that CD could regulate a series of genes to cope with the low-P stress. We found only one common DEG, *Glyma20g33710.2,* in both the roots of CD and YH, with the fold change 0.44 and 0.32 down-regulated in low-P-tolerant accession CD and low-P-sensitive accession YH, respectively.

The number of DEGs in the roots was more than that in the leaves. Interestingly, one common DEG, *Glyma01g04350.1* named as *GmMMP2*, which encodes a matrix metalloproteinase, was found both in the roots and leaves of CD. The fold change is 4.10 and 3.59, respectively. Previous studies demonstrated that it was associated with pathogenic infections in soybean [30].

**Fig. 1:**
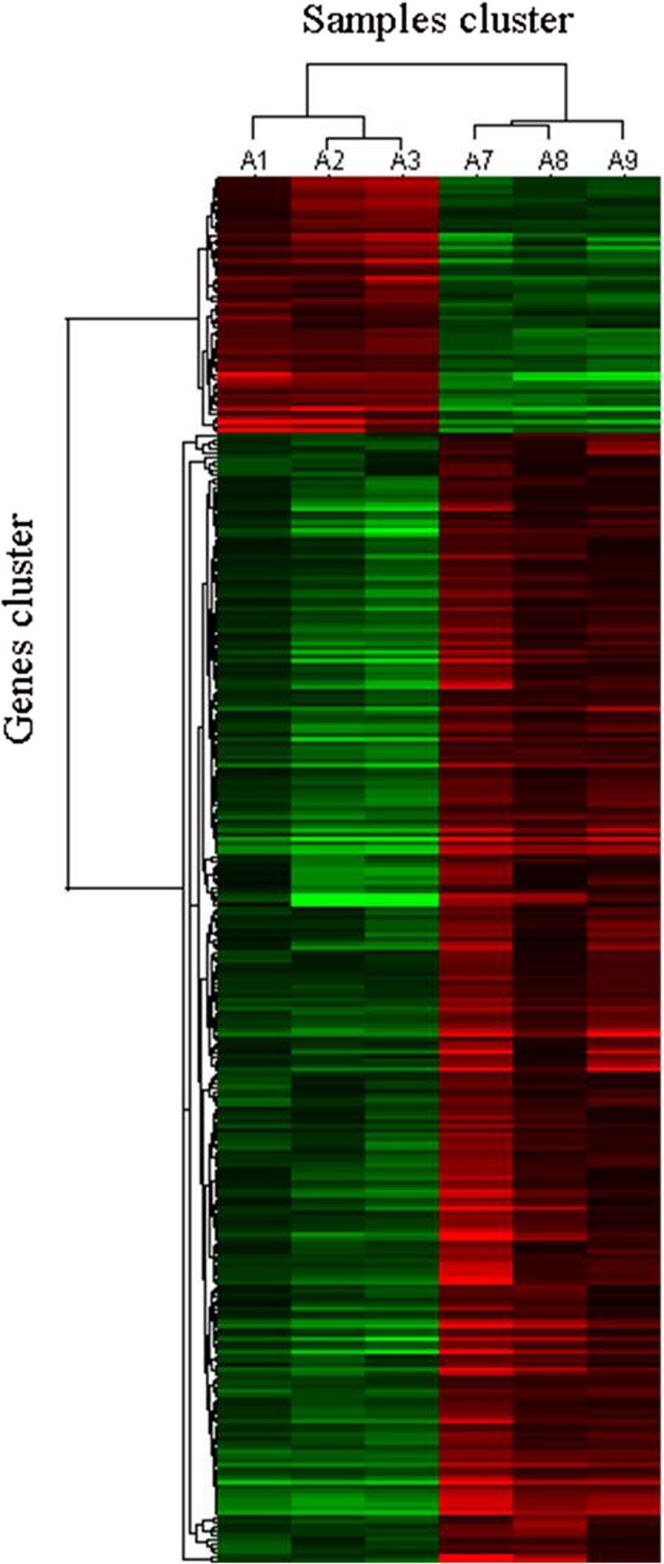
Hierarchical clustering of 319 differentially expressed genes in different P-treated conditions. A1, A2 and A3, three biological replicates of control groups (0.5 mmol/L P); A7, A8 and A9, three biological replicates of treatment groups (0.005 mmol/L P). Red and green indicate high and low expression levels, respectively

A total of 317 non-redundant DEGs were found between treated and control conditions in the roots and leaves of both soybean accessions used in this experiment. The hierarchical clustering analysis classified the tolerant and sensitive soybean accessions into two clusters; samples cluster and genes cluster. The compactness of the clusters of the three replicates confirmed the high reliability of our experimental design (Fig. 1).

To evaluate the potential functions of the DEGs between low-P stress and normal conditions in both soybean accessions, Gene Ontology (GO) categories were applied for low-phosphorus-induced genes. The three GO terms were categorized as biological processes, molecular functions, and cellular components. We found that DEGs were significantly associated with response to wounding, response to jasmonic acid stimulus, oxidoreductase activity, and cell wall, whether they were up-regulated genes or down-regulated genes. Furthermore, we identified pathways that were associated with low-P induced genes with the use of KEGG (Kyoto Encyclopedia of Genes and Genomes). Overall, 45 pathways with *P* values less than 0.05 were motivated under low-P stress (Additional file 2: Table S1), and most pathways were related to metabolic process. Notably, the most significant 3 pathways that were enriched included phenylpropanoid biosynthesis, methane metabolism and phenylalanine metabolism, all related to the metabolism of secondary metabolites.

### Significantly differentially expressed genes between different soybean accessions

Accessions CD and YH showed strong differences in the ability to suffer low-P stress (Additional file 1: Figure S1). There were much more DEGs in CD (309) than in YH (10) (Table 1, Additional file 3: Table S2). To further reveal the DEGs related with low-P condition between CD and YH, we compared the DEGs between soybean accessions CD and YH (Table 2).

**Table 2:** The number of DEGs in CD/YH and roots/leaves

Expression pattern		CD/YH		Roots/leaves	
		Roots	Leaves	CD	YH
Normal P ↑		1325	3835	5945	7631
Low P ↑^a^	Normal P ↑ ^b^	941	2636	5137	6149
	Normal P → ^c^	826 (93) ^e^	975 (40)	1232 (97)	1207 (63)
	Normal P ↓ ^d^	10	1	1	0
	Total	1777	3612	6370	7356
Normal P ↓		1091	1771	5173	4799
Low P ↓	Normal P ↓	610	1040	4696	4156
	Normal P →	324 (14)	526 (38)	893 (19)	1075 (73)
	Normal P ↑	0	0	0	0
	Total	934	1566	5589	5231

^a^ Significantly up-regulated expression pattern under low-P stress;

^b^ Significantly up-regulated expression pattern under normal P condition;

^c^ The expression patterns were not significantly changed under normal P condition;

^d^ Significantly down-regulated expression pattern under normal P condition;

^e^ The number in parentheses indicates the number of genes identified by our criteria (Additional file 4: Figure S2)

DEGs induced by low-P conditions were classified as three categories according to their expression patterns in normal conditions. For 1777 up-regulated genes in roots between CD and YH in low-P stress (Additional file 4: Figure S2), the three categories were as follows: 1) 941 genes were up-regulated in CD/YH in normal conditions, which is consistent with the expression pattern in low-P stress; 2) 10 genes were down-regulated in normal conditions, which showed the opposite expression patterns between normal and low-P conditions; and 3) 826 genes showed no expression difference in normal condition, with the expression difference less than two fold between CD and YH. By the criterion that genes with two fold expression change between experimental group and control group were identified as DEGs, the expression level in CD was more than two fold higher than that in YH for the 1777 up-regulated genes (Additional file 4: Figure S2). Among the 1777 up-regulated genes, there were 826 genes with no expression difference in normal conditions, the expression difference of which between CD and YH was less than two fold. To make the expression difference of CD/YH between low-P stress and normal conditions higher than two fold, we chose those genes with larger than 4-fold expression difference between CD and YH under low-P conditions as candidates. By this criterion, 93 genes with larger than 4-fold expression difference between CD and YH under low-P condition from the 826 genes were chosen as candidates. Hence, we got 40, 14, and 38 DEGs from 975, 324, and 526 DEGs, respectively (Table 2). Combined with the genes with opposite expression patterns, they were considered as marked DEGs between CD and YH. Therefore, 196 marked DEGs were obtained. Among these genes, one gene was up-regulated more than 4 fold between CD/YH under low-P conditions in both roots and leaves. Thus, there were 195 non-redundant marked DEGs detected in total. Among these marked DEGs, the majority (143 genes, 73.33 %) were up-regulated in CD/YH under low-P stress. Additionally, there were 117 and 79 genes expressed differently between CD and YH in roots and leaves, respectively. This result suggested more genes were activated in roots than in leaves under low-P stress during seedling stage.

We performed the enrichment of GO and KEGG analysis for these 195 marked DEGs. GO analysis showed that these genes were mainly involved in secondary metabolic processes, for example flavonoid biosynthesis. They were also involved in response to hormone stimuli (jasmonic acid, auxin, ethylene, and salicylic acid), and defense and abiotic stimuli (oxidative stress, salt stress response, and hypersensitive response). In addition, they were involved in electron carrier activity and peroxidase. KEGG analysis revealed the pathways enriched were mostly associated with the metabolism of secondary metabolites, including phenylpropanoid biosynthesis, methane metabolism, phenylalanine metabolism, flavonoid biosynthesis and so on (Additional file 2: Table S1).

The 317 DEGs in different P-treated conditions were considered as genes in response to low-P stress. The 195 marked DEGs in different soybean accessions were not only induced by low-P stress but also by materials-variation dependent upon the response to low-P conditions. Thus we compared 317 DEGs and 195 marked genes, finding 85 overlapping genes that were considered as active genes induced by low-P stress. Further study revealed most of the common genes focused on roots, which was consistent with a greater number of DEGs in roots than leaves in different P conditions.

Interestingly, the 10 genes that showed opposite expression patterns in roots all belonged to the overlapping genes (Additional file 5: Table S3). Despite two genes (ProbeSetID:11829195 and 12212657) that could not find corresponding symbol and one gene (ProbeSetID:11858123) that could not find functional annotation, seven genes (*GmNAC11* [*Glyma16g04740.1*], *Glyma03g31940.1*, *Glyma08g20220.1*, *Glyma13g27820.1*, *Glyma13g31580.1*, *Glyma18g53170.1* and *Glyma19g32700.1)* with functional annotations were shown to be associated with biotic and abiotic stresses.

Likewise, GO and KEGG enrichment analyses were applied to evaluate the potential functions of the 85 overlapping DEGs. DEGs significantly enriched in GO terms of biological processes such as oxidation reduction and response to jasmonic acid stimulus. Molecular functions such as oxidoreductase activity, and cellular components such as the cell wall, were also enriched. Meanwhile, sixteen pathways were enriched based on 0.05 significance level. Similar to the results of the 195 marked genes, the pathways of phenylpropanoid biosynthesis, methane metabolism, phenylalanine metabolism, and flavonoid biosynthesis were also detected (Additional file 2: Table S1).

### Significantly differentially expressed genes between roots and leaves

In our study, under low-P treatment, more DEGs were detected in roots than leaves, suggesting there were more genes in response to low-P stress during seedling stage in roots. DEGs between roots and leaves were found (Table 2). DEGs induced by low-P conditions were classified into three categories according to their expression patterns in normal conditions. For 6370 up-regulated genes between roots and leaves in CD under low-P stress, three categories of DEGs were 5137 up-regulated genes, one down-regulated gene, and 1232 genes showing no different expression in roots/leaves in normal conditions. Among the 1232 genes, there were 97 genes that showed larger than 4-fold expression difference between roots and leaves under low-P conditions. Additionally, we found 63, 19, and 73 genes showing larger than 4-fold expression differences under low-P conditions from the 1207, 893, and 1075 genes, respectively (Table 2). With one opposite-expressed gene, there were 253 marked DEGs between roots and leaves.

We performed the enrichment of GO and KEGG analysis for these 253 marked DEGs. GO analysis showed these genes enriched in a group of biological processes, for example, oxidation reduction, response to oxidative stress, and response to abiotic stimulus. KEGG analysis revealed most of these genes enriched in methane metabolism, phenylpropanoid biosynthesis, and phenylalanine metabolism (Additional file 2: Table S1).

The 253 marked DEGs between roots and leaves were related with low-P stress. There were 317 DEGs in roots and leaves considered to be caused by low-P stress. Thus, we compared 317 DEGs and 253 marked genes and found 68 overlapping genes. Interestingly, 64 overlapping genes were up-regulated in CD between roots and leaves, consistent with the results that more genes were induced in CD than YH under low-P stress. GO enrichment analysis showed the 68 overlapping genes enriched in biological processes such as response to cadmium ion, defense, jasmonic acid stimulus (*P* < 0.05). In the molecular function term and cellular component, these genes were significantly enriched in transcription regulator activity and in the nucleus (*P* < 0.05) (Additional file 2: Table S1). Furthermore, we identified pathways including methane metabolism and flavonoid biosynthesis (*P* < 0.05).

### Common DEGs among treatments, materials, and tissues

**Fig. 2:**
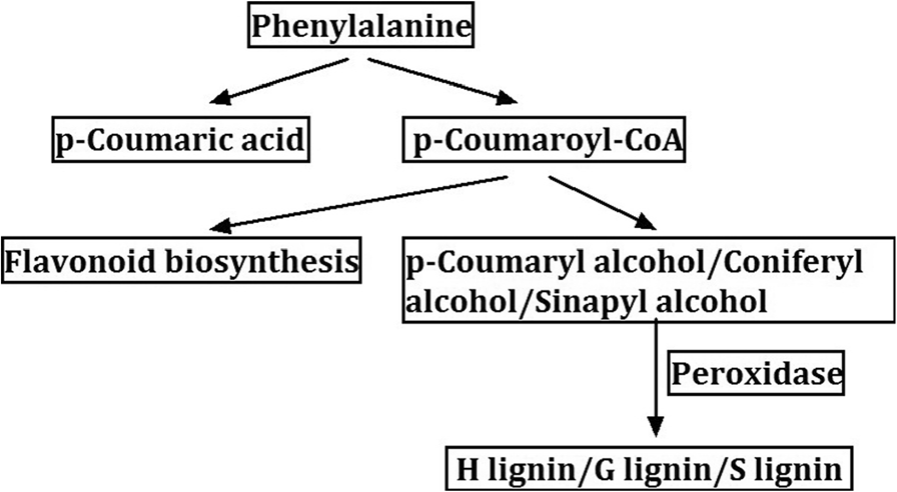
Common DEGs enriched on phenylpropanoid biosynthesis pathway in soybean

The 195 marked DEGs between CD and YH under low-P stress were identified as DEGs between materials which were associated with low-P stress, while the 253 marked DEGs between roots and leaves in low-P conditions were regarded as DEGs between tissues that were related to low-P stress. We compared the 195, 253 marked DEGs with the 317 DEGs between different P treatments and 42 common DEGs were found to be in all three classes. That is to say, the 42 DEGs occurred not only between treatments, but also between materials and tissues under low-P stress, which suggested they were active genes under low-P conditions. Further KEGG analyses enriched in three general pathways: methane metabolism, phenylalanine metabolism, and phenylpropanoid biosynthesis. Notably, the 5 genes enriched in the three pathways were all the same. Among them, four genes including *Glyma02g40000*, *Glyma11g29890*, *Glyma18g06250* and *Glyma20g30910* acted as peroxidase to generate p-Hydroxyphenyl lignin (H lignin), Guaiacyl lignin (G lignin) and Syringyl lignin (S lignin) from corresponding p-Coumaryl alcohol, Coniferyl alcohol and Sinapyl alcohol (Fig. 2). One gene named *Glyma02g14450* functioned in the production of p-Coumaroyl-CoA, which involved in the biosynthesis of flavonoid.

### Quantitative Real-time PCR (RT-qPCR) verification

**Fig. 3:**
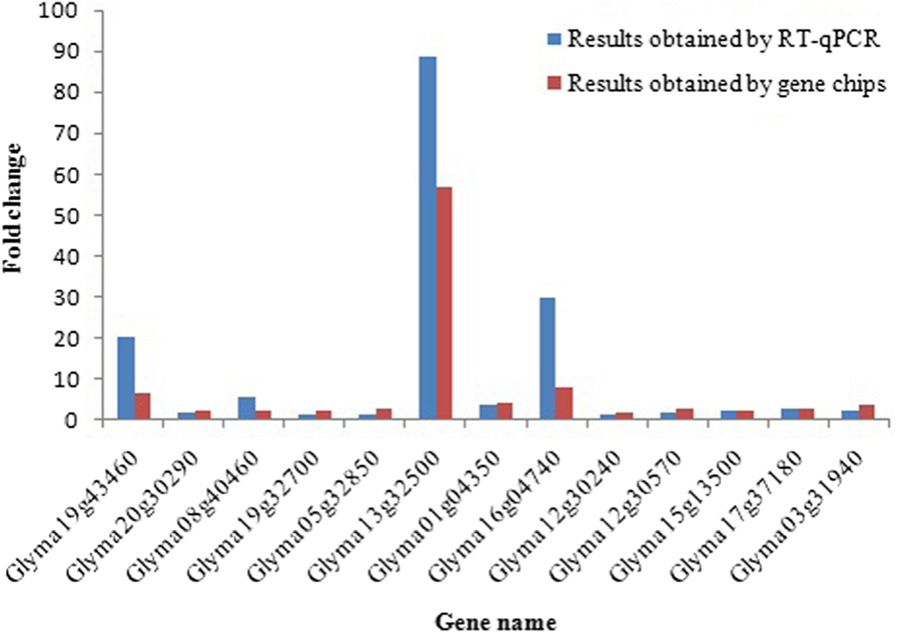
Validation of the results obtained by gene chips with RT-qPCR

To verify the expression results obtained by gene chips, a total of 13 differently expressed genes either between soybean accessions CD and YH in low-P stress or between different P treatments in roots of CD and YH were selected for RT-qPCR. The results of RT-qPCR analysis were consistent with the data obtained from gene chips (Fig. 3), indicating the reliability of the results from gene chips.

## Discussion

### Low phosphorus induced systemic changes in soybean

Phosphorus is important to soybean growth and development. To detect low-P-stress related genes, multiple comparative analyses were conducted. First, we compared the expression level changes between different P treatments in the given materials and tissues, which represented intra-material and intra-tissue DEGs in coping with low-P conditions. Simultaneously, we analyzed inter-materials and inter-tissues gene expression patterns in the case of different concentrations of phosphorus. Finally, we obtained overlapping DEGs between inter-materials and intra-materials, between inter-tissues and intra-tissues, and common genes among different P treatments, materials and tissues. Through these comparative analyses, we acquired stable genes responding to low-P stress, and obtained common genes and pathways related to low-P stress in soybean.

Zeng et al. revealed 17 biological processes in GO enrichment analysis of phosphate-deficiency-responsive genes in soybean roots (*Glycine max* var. Williams 82) [28]. Among these processes, photosynthesis, iron ion transport, fatty acid metabolic process, and stress responses were also detected in our study. Through the analyses of the GO enrichment results, we found oxidative response, as well as peroxidases were repeatedly detected. As earlier studies demonstrated, the expression levels of peroxidases were closely related to the resistance to biotic and abiotic stresses, such as salt stress, wounding, diseases, hypersensitive reactions, and so on [31–34]. Recent studies confirmed that under P-limited conditions, alterations of oxidative stress- related genes including peroxidase genes were observed in *Zea mays*, rice and *Arabidopsis* [23, 24, 27].

Simultaneously, the results of GO enrichment analyses also showed many genes induced by low-P stress were associated with plant hormone signal transduction, such as ethylene and jasmonic acid. Previous studies supported that many genes induced by low-P stress were associated with plant hormone signal transduction [23–25, 35]. An increase of ethylene content in root systems of common bean indicated that ethylene regulated changes in root morphology (i.e. root length) under low-P stress [36]. Meanwhile, the effect of ethylene on the elongation of primary roots in *Arabidopsis* and the emergence of root hairs under low-P stress was shown with the use of a method based on image processing [37]. Gao et al. studied the effect of ethylene on physiological changes of soybean plants grown in P-deficient solution. Their results suggested that ethylene increased root vigor and acid phosphatase activity in the root system, as well as root:shoot ratio under low-P stress [38]. In addition, jasmonic acid promoted plants’ freezing tolerance with the application of exogenous jasmonic acid and blocking-up biosynthesis of jasmonic acid [39].

The three common pathways, methane metabolism, phenylalanine metabolism, and phenylpropanoid biosynthesis, were enriched from comparative analyses, which suggested that these pathways responded actively when plants suffered low-P stress. These results were further supported by the facts that transcript profiling analyses of *Zea mays* and *Arabidopsis* identified that many genes involved in biosynthesis of phenylpropanoids either up-regulated or down-regulated when exposed to low-P stress [24, 25]. Phenylpropanoid was a member of phenolic compound, which pertained to plant secondary metabolites. For example, Hall et al. found that the phenolic content was stimulated by an increase in nutrient stress in *Helianthus annuns* [40]. Similar phenomenon occurred in *Tageteserecta* when suffering from water stress with a significant increase of phenolic content [41]. Koeppe et al. observed that more phenol compounds were leached from living intact roots, dried roots, and tops of phosphate-deficient plants than from phosphate-sufficient ones [42]. Although the effect of methane metabolism on the response to low-P stress had not been clearly stated, a recent study suggested plants released more methane at high temperatures [43], and methane could play an important role in regulating plant growth [44]. Methane metabolism in association with low-P stress may provide a new direction in studying the mechanism of low-P tolerance in soybean.

Our results suggested that the responses of soybean to low-P stress were complex cross-talks; it not only depended on other nutrition (iron) and plant hormones levels (ethylene and jasmonic acid), but also on the metabolism of secondary metabolites. This information can be used to focus on these systemic connections induced by low-P stress to devise strategies aimed at improving soybean yield in P-deficiency soils.

### Massive genes involved in response to low-P stress in low-P-tolerant accession

We selected a low-P-tolerant accession, CD, and a low-P-sensitive accession, YH. The numbers of different expression genes between different P treatments in CD were 30-fold more than those in YH. To explore whether the DEGs were accession-specific between CD and YH, we compared the DEGs between these two accessions. Our results showed that among the 10 DEGs in YH, one gene (*Glyma20g33710*) was also found in CD. A homologous gene was identified in *Lotus japonicus* (Accession No. AB378629) with the tool of BLAST in the National Center for Biotechnology Information (NCBI) database (http://blast.ncbi.nlm.nih.gov/Blast.cgi), which exhibited 54 % sequence identify to *Glyma20g33710*. This gene was predicted as a nodulation-associated bZIP transcription factor gene [45]. For a DEG *Glyma09g18770* in YH, a homologous gene *Glyma17g10510* was detected in the DEGs from CD. *Glyma09g18770* encoded a putative E3 ligase protein with metal ion binding and DNA binding domains, homologous with *BRUTUS* and *OsHRZ* in *Arabidopsis thaliana* and *Oryza sativa* which regulates the response to iron deficiency [46]. The rest annotated DEGs in YH, three genes (*Glyma03g28630*, *Glyma19g31361* and *Glyma03g28611*) encoded the transcription factor ORG2-like proteins. The homologous gene in *Arabidopsis thaliana* was *bHLH038*, which played an important role in the iron-deficiency responses and uptake of this nutrient [47]. *Glyma04g41510* was a plastid-lipid-associated fibrillin protein, transferring phosphorus-containing groups. *Glyma20g33710* encoded a member of basic leucine zipper transcription gene family, homologs with *TGA4* in *Arabidopsis thaliana. TGA4* acts as a regulator in response to defense signals [48]. Although no homologous genes of the rest of the DEGs were detected in CD; gene response to iron-deficiency, uptake, and defense were detected in CD. These genes might be located in different pathways or different locations of the same pathway.

In conclusion, most DEGs were specific between CD and YH (Additional file 3: Table S2), however, the involved pathways found in YH were included in CD. In addition, other low-P stress related pathways were detected in CD, for example, oxidation-reduction and metabolic processes. The much more genes induced by low-P stress existed in CD than in YH suggested massive genes involved in the response to low-P condition in tolerant accession CD. In addition to involving in biotic and abiotic stress responses, some of these genes were related to plant growth and development. On one hand, these massive genes could response to low-P condition through mediating the secretion of phytohormone and secondary metabolites. On the other hand, they could improve uptake of P through controlling soybean root traits, such as main root elongation and increased root hair number. All of these responses could make CD more adaptive to low-P stress and more resistant than YH. Likewise, more stress-responsive genes were identified in tolerant soybean line than those in sensitive soybean line in the response to common cutworm feeding [49]. Moreover, more DEGs were detected in CD than those in YH under low-P stress (Table 1) could be due to different genetic backgrounds between these two materials, which was further supported by the result that there were 64,534 SNPs detected between CD and YH by a genome-wide NJAU 355 K SoySNP array (unpublished data). The selection of P-efficient soybean accessions could be helpful in exploring low-P related genes and creating new low-P-tolerant soybean accessions.

### Roots played a crucial role in P metabolism during seedling stage

Compared with 310 DEGs detected in roots under low-P stress, only 18 DEGs were detected in leaves (Table 1). Plants under low-P conditions depended on the root system during seedling stage to absorb phosphorus nutrition. Previous studies showed an increase in lateral roots, root hair density, root:shoot ratio when plants were exposed to low-P stress [50, 51]. Among them, uptake through root hairs contributed up to 90 % of the phosphorus acquired by plants [52]. Some phosphate transporters specifically located in roots had been identified to be involved in Pi uptake at the root-soil interface in P-deficient soils. These proteins transport available phosphorus that could be absorbed by plants, mainly H_2_PO_4_^−^, HPO_4_^2−^and PO_4_^3−^ [53]. The apparent changes of root systems under low-P stress in our hydroponic experiments also confirmed that roots played an important role in acquiring P during seedling stage. As such, we found some genes associated with phosphorus absorption in roots. The gene *Glyma04g19450* showed homology with *AtSPX2-4*, members of a sub-family of *Arabidopsis* genes with the SPX domain. Proteins harboring SPX domain are believed to be involved in phosphorus acquisition and phosphorus signaling network. Duan et al. found several Pi starvation-responsive genes were regulated by *AtSPX2-4*, positively or negatively. Further studies demonstrated the expression levels of *AtSPX2* and *AtSPX3* increased under phosphorus starvation [10]. Interestingly, we also found three DEGs in roots; namely *Glyma13g24810.1*, *Glyma07g31630.1* and *Glyma13g31290.1* respectively, showed homology with *PHO2*, the ubiquitin-conjugating enzyme that contained SPX domain, which is crucial for phosphorus acquisition and translocation in plants. Results of Huang et al. suggested *PHO2* modulated Pi absorption, specifically transport at the root surface by regulating the abundance of PHT1s [54].

The limited number of DEGs in leaves probably resulted from genes in the leaves primarily involved in P distribution and utilization in later stages. Three DEGs from leaves (*Glyma03g28630*, *Glyma19g31361* and *Glyma03g28611*) were homologous to *bHLH038* in *Arabidopsis thaliana*, functioning in the iron-deficiency responses and uptake [55]. Thus, we concluded roots played an important role in coping with low-P stress during seedling stage. An in-depth study on DEGs in roots will advance the functional study of highly P-efficient genes.

## Conclusion

P starvation leads to systemic changes in the gene expression of soybean. Here, the gene expression patterns of soybean under low-P stress were surveyed on the roots and leaves of a low-P-tolerant accession and a low-P-sensitive accession by microarray chips. Through comparative analyses on the differently expressed genes, we identified 42 candidate genes and three common pathways, including methane metabolism, phenylalanine metabolism and phenylpropanoid biosynthesis, which were highly correlated to low-P stress. These results not only promote our understandings of the molecular bases of the responses to P deficiency, but also facilitate research in improving Pi usage in soybean and designing highly phosphate-efficient soybeans. This process could optimize fertilizer use and promote development of sustainable agricultural practices.

## Methods

### Plant materials

Through hydroponic experiments, we selected two soybean accessions, Chundou (CD) and Yunhefengwodou (YH), from 219 soybean materials [29]. The 219 soybean seedlings were sown in plastic pots which contained the nutrient soil and vermiculite with a ratio of 1:3. After five days of germination, we picked three shoots of each accession with similar growth vigor which were then transplanted to half Hoagland under different P treated conditions in hydroponic boxes with a 16 h/8 h (day/night) photoperiod and a temperature of 26-28 °C/22 °C (day/night) temperature cycle. Hoagland nutrient solution was composed of macroelements (1.0 mM KH_2_PO_4_, 5.0 mM KNO_3_, 5.0 mM Ca(NO_3_)_2_, 2.0 mM MgSO_4_), microelements (2.86 mg/L H_3_BO_3_, 1.81 mg/L MnCl_2_ · 4H_2_O, 0.22 mg/L ZnSO_4_ · 7H_2_O, 0.08 mg/L CuSO4 · 5H_2_O, 0.0269 mg/L Na_2_MoO_4_ · 2H_2_O) and ferric salts (5.56 mg/L FeSO_4_ · 7H_2_O, 7.64 mg/L EDTA · Na). Hoagland with 1.0 mmol/L P was considered as + P treated condition, while Hoagland with 0.01 mmol/L P was considered as -P treated condition. To satisfy the demands of plant growth, we substituted equal concentrations of KCl for KH_2_PO_4_. Ventilation was performed three times per day for 30 min. Nutrient solution was exchanged every three days.

Then Roots and leaves of soybean accession CD and YH were harvested after 10 days of hydroponics (normal and low P treated conditions), each with three biological replicates. All samples were stored at −80 °C for additional experiments, including RNA isolation and Microarray experiments.

### Total RNA isolation and quantitative RT-PCR

Total RNA was extracted from roots and leaves separately of soybean accessions CD and YH, each with three biological replicates. A total of 24 samples were isolated to exact RNA according to the manufacturer’s instructions with the use of Plant RNA Extract Kit (TianGen, Beijing, China). A total of 50–100 mg leaf and 50–100 mg root were used to isolate RNA for each sample, respectively. Then about 1 μg RNA was used to configure 20 μL system to synthesize cDNA with the application of HiScript® II Q RT SuperMix for qPCR (+gDNA wiper) (Vazyme, Nanjing, China). The constitutive expression gene *Gmtublin* (GenBank accession number: AY907703) was used as a reference gene for RT-qPCR [56], and each sample was measured with three replicates. The RT-qPCR was conducted on an ABI 7500 real-time PCR system (Applied Biosystems, Forster City, CA, USA) with the use of SYBR Green Realtime Master Mix (Toyobo). The ABI 7500 system Sequence Detection System (SDS) software v.1.4 was applied to analyze the data. The primers used were listed in Additional file 6: Table S4.

### Microarray experimental design and data analysis

To obtain the differently expressed genes under different P treated conditions in different soybean accessions, we used 24 Affymetrix Soybean Gene 1.1 ST Array Strip. These detected 66,473 genes from Glyma1.01 database, each with 19 probes, and 8250 genes from GeneBank database, each with 16 probes. After RNA concentrations quantified by ultraviolet Spectrophotometer (NanoDrop Technologies, ND-1000) and RNA quality assessed by formaldehyde agarose gel electrophoresis, the total mRNA was hybridized on Affymetrix Soybean Gene 1.1 ST array strips. Microarray experiments were performed by CapitalBio Technology. All microarrays were scanned with an Affymetrix scanner named GeneChip® Scanner 3000, and images acquired were saved as .JPG pictures. AGCC software (Affymetrix®GeneChip® Command Console® Software) was applied to convert the image signals to digital signals, which recorded the signal intensity of every probe. After subtracting background and consolidating probe signals, normalization between arrays was carried out using RMA algorithm to remove variances between samples caused by abiologic factors [57]. We compared gene expression quantities between different P-treated conditions and different soybean accessions. Differently expressed genes simultaneously satisfied all of the following criteria: (a) |log_2_Ratio| ≥ 1, ratio represented fold change of expression between experimental group control group, (b) the P value after FDR correction was less than 0.05, (c) three biological replicates existed. The analyses of differently expressed genes were performed using SAM (significance analysis of microarray). For DEGs between different P treatments, A1, A2 and A3 were defined as three biological replicates under normal condition (0.5 mmol/L P), and A7, A8 and A9 were defined as three biological replicates under low-P condition (0.005 mmol/L P) (Fig. 1).

### Gene Ontology (GO) and KEGG pathway enrichment analysis

Gene Ontology database was a structured typical biological model; it consisted of three terms which were cellular component (CC), biological process (BP) and molecular function (MF). All differently expressed genes were mapped to GO terms in the GO databases (http://www.geneontology.org/), then calculated GO terms that were significantly enriched in differently expressed genes compared with genome background with the application of hypergeometric distribution. Results showed biological functions that were significantly associated with genes (*P* < 0.05).

The main metabolic pathways and signal transduction pathways that differently expressed genes may be identified by pathway enrichment analysis. KEGG (Kyoto Encyclopedia of Genes and Genomes) database is an important public database associated with pathways which integrateds genomics, biochemistry and system functional omics. Using KEGG PATHWAY as a unit, we found pathways that were significantly associated with differently expressed genes compared with genome background. Expression profile data were analyzed using a Molecule Annotation System (http://mas.capitalbiotech.com/).

### Availability of supporting data

The data sets supporting the results of this article are included within the article and its additional files. The data from the 24 chips are publicly available in the National Center for Biotechnology Information (NCBI) Gene Expression Omnibus (GEO) under accession number GSE78242 (http://www.ncbi.nlm.nih.gov/geo/query/acc.cgi?acc=GSE78242).

## Additional files

Additional file 1: Figure S1. Roots morphological of soybean accession CD and YH under low-P condition (0.005 mmol/L P) (A) Root morphological before low-P treatments (B) Root morphological after 10 days of low-P treatments. (DOC 190 kb) Additional file 2: Table S1. KEGG and Go enrichment analysis of differently expressed genes. (a) 317 DEGs between different P treatments (b) 195 DEGs between different materials (c) 253 DEGs between different tissues (d) 85 overlapping genes between the 195 marked DEGs between CD and YH and 317 DEGs in roots and leaves (e) 68 overlapping genes between the 253 marked DEGs between roots and leaves and 317 DEGs in roots and leaves (f) 42 common DEGs. (XLS 86 kb) Additional file 3: Table S2. The list of DEGs between different P treatments in the roots and leaves of CD and YH. (XLS 55 kb) Additional file 4: Figure S2. Identification of differently up-regulated genes in roots between different soybean accessions. (DOC 26 kb) Additional file 5: Table S3. 10 genes showing opposite expression patterns in roots between different soybean accessions. (DOC 42 kb) Additional file 6: Table S4. The list of primers used in this article. (DOC 32 kb)

## Abbreviations

BP: biological process
CC: cellular component
CD: Chundou
DEGs: Differently expressed genes
GO: Gene Ontology
KEGG: Kyoto Encyclopedia of Genes and Genomes
MF: molecular function
P: phosphorus
QTL: quantitative trait loci
RT-qPCR: Quantitative Real-time PCR
SDS: Sequence Detection System
YH: Yunhefengwodou
