# Supplementary material for: A genome-wide expression profile analysis reveals active genes and pathways coping with phosphate starvation in soybean
Source: BMC Genomics. 2016 Mar 5;17:192. doi: 10.1186/s12864-016-2558-9 (PMC4779269; doi:10.1186/s12864-016-2558-9)
Supplement: Additional file 1: Figure S1. — Roots morphological of soybean accession CD and YH under low-P condition (0.005 mmol/L P) (A) Root morphological before low-P treatments (B) Root morphological after 10 days of low-P treatments. (DOC 190 kb) [file 12864_2016_2558_MOESM1_ESM.doc]

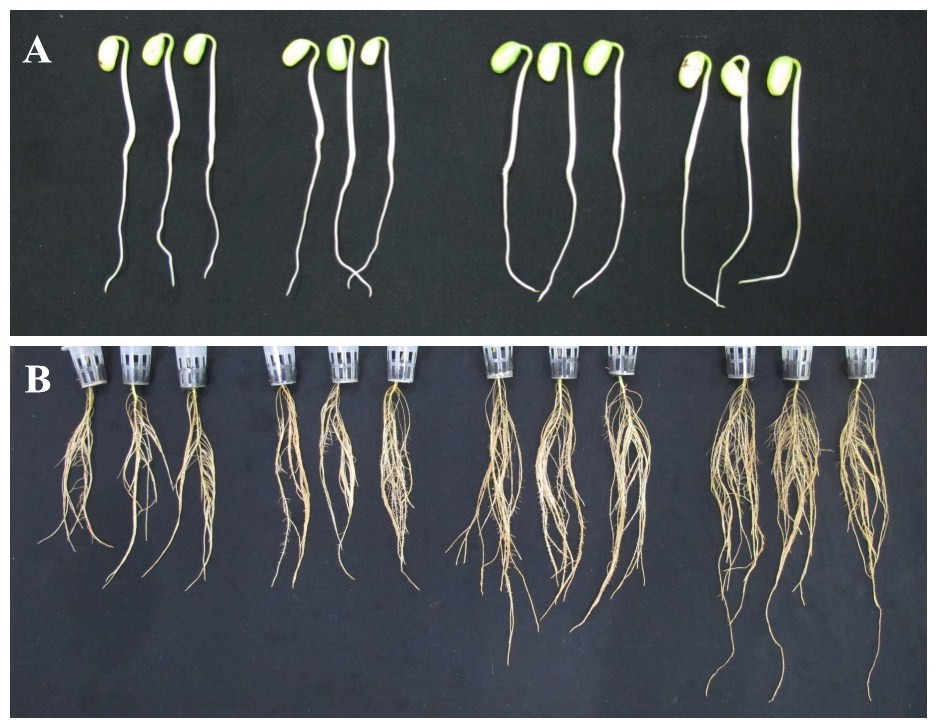


**YH(+P) CD(+P) YH(-P) CD(-P)**

**Additional file 1: Figure S1.** Roots morphological of soybean accession CD and YH under low-P condition (0.005 mmol/L P) (A) Root morphological before low-P treatments (B) Root morphological after 10 days of low-P treatments.
