# Supplementary material for: A genome-wide expression profile analysis reveals active genes and pathways coping with phosphate starvation in soybean
Source: BMC Genomics. 2016 Mar 5;17:192. doi: 10.1186/s12864-016-2558-9 (PMC4779269; doi:10.1186/s12864-016-2558-9)
Supplement: Additional file 4: Figure S2. — Identification of differently up-regulated genes in roots between different soybean accessions. (DOC 26 kb) [file 12864_2016_2558_MOESM4_ESM.doc]

Normal P ↑ 1325

Low P ↑1777 ^a^

Low P ↑ Normal P ↓ 10

Low P ↑ (>4 fold) Normal P → (<2 fold) 93 ^c^

Low P ↑(>2 fold) Normal P → (<2 fold) 826 ^b^

Low P ↑ Normal P ↑ 941

**Additional file 4: Figure S2.** Identification of differently up-regulated genes in roots between different soybean accessions

^a^ In low-P stress, 1777 significantly up-regulated genes existed in roots in CD comparing with YH;

^b^ 826 genes showed no expression difference in normal condition, the expression difference of which was less than two fold, while these genes were significantly up-regulated under low-P stress;.

^c^ 93 genes showed no expression difference in normal condition but showed larger than 4-fold expression difference between CD and YH under low-P condition.
