# Supplementary material for: A genome-wide expression profile analysis reveals active genes and pathways coping with phosphate starvation in soybean
Source: BMC Genomics. 2016 Mar 5;17:192. doi: 10.1186/s12864-016-2558-9 (PMC4779269; doi:10.1186/s12864-016-2558-9)
Supplement: Additional file 5: Table S3. — 10 genes showing opposite expression patterns in roots between different soybean accessions. (DOC 42 kb) [file 12864_2016_2558_MOESM5_ESM.doc]

**Additional file 5: Table S3.** 10 genes showing opposite expression patterns in roots between different soybean accessions

| ProbeSetID | Symbol-traID | fold changes | | functional annotation | homologous genes | references |
| --- | --- | --- | --- | --- | --- | --- |
|  |  | down-regulated | up-regulated |  |  |  |
| 11816743 | Glyma03g31940.1 | 0.4651 | 3.9942 | ethylene-responsive transcription factor 15-like | *AtERF15* overexpression lines exhibited salt and drought tolerance | 1 |
| 11829195 | -- | 0.2988 | 4.6541 | -- |  |  |
| 11858123 | Glyma05g22760.1 | 0.4928 | 4.3266 | -- |  |  |
| 11930922 | Glyma08g20220.1 | 0.4634 | 2.301 | seed linoleate 9S-lipoxygenase-like | *LOX* gene expression was modulated in response to water deficit and wounding | 2 |
| 12052020 | Glyma13g27820.1 | 0.2911 | 5.1946 | basic 7S globulin-like | Expression level was associated with heat stress in soybean | 3 |
| 12067310 | Glyam13g31580.1 | 0.2528 | 2.159 | stem-specific protein TSJT1-like |  |  |
| 12124458 | Glyma16g04740.1 | 0.328 | 3.5663 | transcriptional factor NAC11 | Play important roles in dealing with multiple stress and it regulated the expression of stress-response genes, such as *DREB1A* and *EDR11* | 4 |
| 12165516 | Glyma18g53170.1 | 0.2192 | 2.2763 | putative glutamine amidotransferase-like protein  RP404-like | contained a conserved domain that is associated with aluminium |  |
| 12193748 | Glyma19g32700.1 | 0.4905 | 2.2219 | polygalacturonase inhibitor-like (PGIPs) | Two PGIP-encoding genes ( *Bnpgip1* and *Bnpgip2*) from *Brassica napus* DH12075 were both induced by jasmonic acid and cold | 5 |
| 12212657 | -- | 0.156 | 5.439 | -- |  |  |

References：

1. Lee SB, Lee SJ, Kim SY: **AtERF15 is a positive regulator of ABA response**. *Plant cell reports* 2015, **34**(1):71-81.

2. Bell E, Mullet JE: **Lipoxygenase Gene-Expression Is Modulated in Plants by Water Deficit, Wounding, and Methyl Jasmonate**. *Mol Gen Genet* 1991, **230**(3):456-462.

3. Fujiwara K, Cabanos C, Toyota K, Kobayashi Y, Maruyama N: **Differential expression and elution behavior of basic 7S globulin among cultivars under hot water treatment of soybean seeds**. *Journal of bioscience and bioengineering* 2014, **117**(6):742-748.

4. Hao YJ, Wei W, Song QX, Chen HW, Zhang YQ, Wang F, Zou HF, Lei G, Tian AG, Zhang WK *et al*: **Soybean NAC transcription factors promote abiotic stress tolerance and lateral root formation in transgenic plants**. *The Plant journal : for cell and molecular biology* 2011, **68**(2):302-313.

5. Li RG, Rimmer R, Yu M, Sharpe AG, Seguin-Swartz G, Lydiate D, Hegedus DD: **Two Brassica napus polygalacturonase inhibitory protein genes are expressed at different levels in response to biotic and abiotic stresses**. *Planta* 2003, **217**(2):299-308.
