# Supplementary material for: A genome-wide expression profile analysis reveals active genes and pathways coping with phosphate starvation in soybean
Source: BMC Genomics. 2016 Mar 5;17:192. doi: 10.1186/s12864-016-2558-9 (PMC4779269; doi:10.1186/s12864-016-2558-9)
Supplement: Additional file 6: Table S4. — The list of primers used in this article. (DOC 32 kb) [file 12864_2016_2558_MOESM6_ESM.doc]

**Additional file 6: Table S4.** The list of primers used in this article

| Gene ID | Primer Sequence (5'to3') of forward chain | Primer Sequence (5'to3') of reverse chain |
| --- | --- | --- |
| *Glyma19g43460* | AACACGGGTGGGACTTGAAC | CAGAAAGCGGTCCAGCCATA |
| *Glyma20g30290* | CGAGAATATGCCACCTAAGATCTG | GGTCACCAATCTCATTAAACAAAAAA |
| *Glyma08g40460* | CCCTGAAATCCAAATCCGAAT | CGTGTGTATGTTGTTGTGTGATAAAAG |
| *Glyma19g32700* | TGCAGCAGAGAATACGTGCAT | GCTTTACAAAGAGGGATGGAAGTAA |
| *Glyma05g32850* | CAGCCTTGGGCTTCGATTT | TCTTGCCACGGTGAGAGATG |
| *Glyma13g32500* | TGCAACAATAATAACACCACCAATT | AAAAACACACAGGGAACATCCTTT |
| *Glyma01g04350* | GGAAAAGCCACTCATGAATCAGT | GGGTCTTCATCCACTAGTTGCAT |
| *Glyma16g04740* | TTTATTTAGCTAGCTAGCCAAT | TGGCCTTAGCTGGTAAGTC |
| *Glyma12g30240* | GCTGAGGTTGCAGGACTTGAA | GACCAATATTACGGGCCACTTG |
| *Glyma12g30570* | CCCCCAGTAAAAGGGAAGATG | CAAATGCCCAATAGCCAGAAA |
| *Glyma15g13500* | ATACGAAAGAGAGAGTGAAGCATCGT | CCAGCCTTTATTTATAGACCCAATTC |
| *Glyma17g37180* | GCAATCGAACCGCGAATC | GATCATCCAAGTGCTTCTGCTTT |
| *Glyma03g31940* | GACTTTGACTTCTTAATCAGCCTCTCT | GAAGGAAGAAGTAGGAACAGAATATAACG |
| *Tublin* | GGAGTTCACAGAGGCAGA | CACTTACGCATCACATAGCA |
